# Most deprived Louisiana census tracts have higher hepatocellular carcinoma incidence and worse survival

**DOI:** 10.3389/fonc.2024.1331049

**Published:** 2024-02-06

**Authors:** Kendra L. Ratnapradipa, Tingting Li, Mei-Chin Hsieh, Laura Tenner, Edward S. Peters

**Affiliations:** ^1^ Department of Epidemiology, College of Public Health, University of Nebraska Medical Center, Omaha, NE, United States; ^2^ Louisiana Tumor Registry, Epidemiology Program, School of Public Health at Louisiana State University (LSU) Health Sciences Center-New Orleans, New Orleans, LA, United States; ^3^ Department of Internal Medicine, Division of Oncology/Hematology, College of Medicine, University of Nebraska Medical Center, Omaha, NE, United States

**Keywords:** Area Deprivation Index (ADI), hepatocellular carcinoma (HCC), incidence, social determinants of health, survival

## Abstract

**Background:**

Liver cancer incidence increased in the US from 1975 through 2015 with heterogeneous rates across subpopulations. Upstream or distal area-level factors impact liver cancer risks.

**Objective:**

The aim of this study was to examine the association between area-level deprivation and hepatocellular carcinoma (HCC) incidence and survival. We also explored the association between area deprivation and treatment modalities.

**Methods:**

Louisiana Tumor Registry identified 4,151 adult patients diagnosed with malignant HCC from 2011 to 2020 and linked residential address to census tract (CT)-level Area Deprivation Index (ADI) categorized into quartiles (Q1 = least deprived). ANOVA examined the association between ADI quartile and CT age-adjusted incidence rate (AAIR) per 100,000. Chi-square tested the distribution of demographic and clinical characteristics across ADI quartiles. Kaplan–Meier and proportional hazard models evaluated survival by deprivation quartile.

**Results:**

Among the 1,084 CTs with incident HCC, the average (SD) AAIR was 8.02 (7.05) HCC cases per 100,000 population. ADI was observed to be associated with incidence, and the mean (SD) AAIR increased from 5.80 (4.75) in Q1 to 9.26 (7.88) in Q4. ADI was also associated with receipt of surgery (*p* < 0.01) and radiation (*p* < 0.01) but not chemotherapy (*p* = 0.15). However, among those who received chemotherapy, people living in the least deprived areas began treatment approximately 10 days sooner than those living in other quartiles. Q4 patients experienced the worst survival with a median of 247 (95% CI 211–290) days vs. Q1 patients with a median of 474 (95% CI 407–547) days (*p* < 0.0001). Q4 had marginally poorer survival (HR 1.20, 1.05–1.37) than Q1 but the association became non-significant (HR 1.12, 0.96–1.30) when adjusted for rurality, liquor store density, sex, race/ethnicity, age, insurance, BMI, stage, hepatitis diagnosis, and comorbidities.

**Conclusion:**

Increasing neighborhood (CT) deprivation (ADI) was observed to be associated with increased HCC incidence and poorer HCC survival. However, the association with poorer survival becomes attenuated after adjusting for putative confounders.

## 1 Introduction

An estimated 41,210 new liver cancer cases will be diagnosed in the United States (US) during 2023, accounting for 2.1% of all incident cancers (1). An estimated 29,380 people in the US will die from liver cancer in 2023, accounting for 4.8% of cancer mortalities (1). The 5-year relative survival rate is only 21.6% (1), which may be partially attributable to a lack of general screening guidelines and a lack of symptoms in early disease stages, potentially leading to delayed diagnosis and more advanced cancer. US liver cancer incidence rates increased from 1975 through 2015 for both men and women before beginning to decrease for men younger than 50 and plateauing for older men. However, rates have continued to increase for both younger and older women (2). Liver cancer incidence exhibits birth cohort effects (3), and risk profiles differ by age group, sex, and race/ethnicity.

Known risk factors for liver cancer include viral hepatitis B (HBV) and hepatitis C (HCV) infections, alcohol use, fatty liver disease, and metabolic syndrome (4). These often co-occur and function synergistically to increase liver cancer risk (4). The modifiable behaviors contributing to liver cancer risk do not occur in a vacuum and are shaped by the environment in which people live. For example, neighborhood disadvantage and convenience store concentration are associated with increased tobacco use (5), and neighborhoods with a mostly Black population and lower neighborhood socioeconomic status (nSES) have a greater concentration of alcohol retailers (6).

Social determinants of health (SDOH) are the conditions under which people are born, grow, live, work, and age. These conditions refer to non-medical factors influencing health including knowledge, attitudes and beliefs, behaviors (e.g., smoking and alcohol consumption), and resource availability. SDOH directly impact the health of individuals and populations; they also help structure lifestyle choices and behaviors, which interact to produce health or disease. At the same time, SDOH are shaped by public policy and thus are theoretically modifiable. Upstream or distal SDOH refer to the factors that comprise structural influences on health and health systems, government policies, and the social, physical, economic, and environmental factors that determine health (7–9).

Contextual-level SDOH include area-level measures of disadvantage, such as the Area Deprivation Index (ADI). Residing in a disadvantaged neighborhood has been associated with higher rates of disease including cardiovascular disease and diabetes, as well as increased health service utilization and premature mortality (8). Multiple indices have been developed to measure the effects of nSES on cancer (10), with nuanced differences in terms of what is being measured and how the index theoretically may represent an array of upstream factors associated with negative health outcomes. Prior studies of hepatocellular carcinoma (HCC) observed that neighborhood disadvantage was associated with increased incidence in Louisiana (11), and the most deprived neighborhoods had higher risk of incident HCC in Texas (12). Additionally, higher county-level social vulnerability has been associated with reduced access to surgical intervention for early-stage HCC for Black and Hispanic populations (13). Neighborhood-level factors impacting risk and treatment access have direct implications for survival disparities. Therefore, this study aimed to examine the association between area-level deprivation and incident HCC in Louisiana with more recent data and using a different measure of nSES. We also sought to examine the association between area-level deprivation and treatment, as well as the association between ADI and HCC survival. Because Louisiana’s population is predominantly White (62.5%) and Black (32.8%) (14), we limited analysis to these two races.

## 2 Materials and methods

This study involving human subjects was reviewed and approved by the institutional review board of the Louisiana State University Health Sciences Center - New Orleans to use Louisiana Tumor Registry data for this study. Written informed consent for participation was not required for this study in accordance with the national legislation and institutional requirements.

### 2.1 Data sources and eligibility

The Louisiana Tumor Registry has a state-wide catchment area and is one of the Surveillance, Epidemiology, and End Results (SEER) cancer registries. We identified all incident cases aged 18 and older diagnosed with primary malignant HCC (primary site code ICD-O-3 code of C220 with histology codes 8170-8175, 8180), between 1 January 2011 and 31 December 2020 who identified as White or Black, regardless of Hispanic ethnicity. US Census and American Community Survey (2013–2017) estimates were used to calculate population denominators for incidence and to calculate the ADI.

### 2.2 Variables

Our outcomes of interest were HCC age-adjusted incidence rates (AAIR) and cause-specific survival time calculated as days from HCC diagnosis to the date of last follow-up or death from cancer. Individuals who died of causes other than cancer were considered censored at the time of death. Our primary exposure variable was ADI, computed at the census tract (CT) level based on cases’ residence at time of diagnosis. Previous studies reported that the census tract is a reasonable geographic level for estimating neighborhood effects, as it tends to represent social and economically homogeneous groups of approximately 4,000–7,000 people (15). The ADI is a weighted composite of 17 census measures of area-level socioeconomic status related to demographics, education, employment, housing, income/poverty, and mobility (16). ADI was converted to quartiles (Q1 = least deprived, Q4 = most disadvantaged neighborhoods).

Treatment modality was classified as surgery, radiation, and chemotherapy, with each coded as yes/no. Time to treatment was defined as date of diagnosis to date of treatment initiation.

Other variables that we considered as confounders or important predictors were as follows: urban (yes and no) defined by the US Census Bureau’s Urban Rural Indicator Codes with urban including codes 1–2 (all and mostly urban) and rural defined as codes 3–4 (mostly and all rural); liquor store density was defined by number of liquor stores per 1,000 population at the CT level by using data from the National Neighborhood Data Archive (NaNDA); sex (male and female); age group (<50, 50–59, 60–69, 70–79, and ≥80); race/ethnicity (non-Hispanic White, non-Hispanic Black, and Hispanic); insurance (private, Medicaid only, other insured including Medicare, unknown insurance, and uninsured); smoking status (never, current, former, and unknown); body mass index (BMI; underweight, normal weight, overweight, obese, and unknown); diagnostic stage (local, regional, distant, and unknown); Charlson Comorbidity Index (0, 1, and ≥2); and hepatitis B or C diagnosis (yes and no) based on the patients’ comorbid condition at the time cancer was diagnosed and treated. We enhanced the hepatitis diagnosis information by linking with Louisiana statewide hospital in-patient discharge data (HIDD). HCC patients with HBV or HCV diagnosis prior to or within 6 months of cancer diagnosis found from the HIDD database were categorized as “yes”.

### 2.3 Statistical analysis

All analyses were performed using SAS statistical software version 9.4 (SAS Institute Inc). ANOVA examined the association between CT ADI and AAIR, reported as quartile average with standard deviation (SD). Chi-square tests evaluated the distribution of demographic and clinical characteristics across neighborhood deprivation level. Kaplan–Meier tests examined median days of survival by ADI quartile. Cox proportional hazard tests evaluated survival outcomes (time of death) with unadjusted and adjusted models. The fully adjusted model used block entry of all covariates to examine the influence of multiple predictors simultaneously, including ADI quartiles, hepatitis status, rural/urban status, liquor stores (per 1,000 people), gender, race/ethnicity, age, health insurance primary payer, smoking status, BMI, stage, and Charlson Comorbidity Index.

## 3 Results

We identified 4,151 eligible cases residing in 1,084 of Louisiana’s 1,148 CTs. **Table 1** summarizes the case baseline characteristics overall and by ADI quartile. The average age at diagnosis was 64.1 (SD 9.5). Most patients resided in urban areas (87.7%), were male (80.2%), identified as non-Hispanic White (58.8%), and had a primary payer other than private insurance or Medicaid (54.3%). Most cancers were diagnosed at a localized stage (50.9%) and occurred in people who had a history of hepatitis B/C infection (52.3%). The most vs. least deprived CTs had higher percentages of cases who were non-Hispanic Black people (*p* < 0.0001), younger than 70 (*p* < 0.0001), Medicaid-insured (*p* < 0.0001), current smokers (*p* < 0.0001), and underweight/normal weight (*p* = 0.0001). Furthermore, CTs with higher deprivation had a higher percentage of cases diagnosed with hepatitis B/C (*p* < 0.0001).

**Table 1:** Baseline characteristics and treatment of Louisiana adults diagnosed with hepatocellular carcinoma between 2011 and 2020 by census tract Area Deprivation Index quartile.

Characteristics	Overall *N* (%)		Q1 (least deprived) *N* = 891 (21.5%)		Q2 *N* = 1,144 (27.6%)		Q3 *N* = 1,115 (26.9%)		Q4 (Most deprived) *N* = 1,001 (24.1%)		*p*-value
**Liquor store density per 1,000 people (mean, SD)**	0.07	0.2	0.08	0.2	0.03	0.1	0.05	0.1	0.13	0.3	**<0.0001**
**Rural/urban status**											**<0.0001**
Urban	3,640	87.7	887	99.6	1,067	93.3	942	84.5	744	74.3	
Rural	511	12.3	4	0.5	77	6.7	173	15.5	257	25.7	
**Gender**											0.09
Male	3,328	80.2	689	77.3	935	81.7	895	80.3	809	80.8	
Female	823	19.8	202	22.7	209	18.3	220	19.7	192	19.2	
**Race/Ethnicity**											**<0.0001**
Non-Hispanic White	2,441	58.8	677	76.0	805	70.4	601	53.9	358	35.7	
Non-Hispanic Black	1,635	39.4	187	21.0	317	27.7	497	44.6	634	63.4	
Hispanic	75	1.8	27	3.0	22	1.9	17	1.5	9	0.9	
**Age**											**<0.0001**
<50	141	3.4	23	2.6	47	4.1	37	3.3	34	3.4	
50–59	1,194	28.8	229	25.7	308	26.9	321	28.8	336	33.6	
60–69	1,765	42.5	358	40.2	491	42.9	472	42.3	444	44.4	
70–79	762	18.4	189	21.2	216	18.9	217	19.5	140	14.0	
≥80	289	7.0	92	10.3	82	7.2	68	6.1	47	4.7	
**Primary Payer**											**<0.0001**
Private insurance	667	16.1	171	19.2	201	17.6	166	14.9	129	12.9	
Medicaid	773	18.6	131	14.7	204	17.8	207	18.6	231	23.1	
Insured other	2,255	54.3	486	54.6	624	54.6	619	55.5	526	52.6	
Unknown	262	6.3	69	7.7	65	5.7	65	5.8	63	6.3	
Not Insured	194	4.7	34	3.8	50	4.4	58	5.2	52	5.2	
**Smoking status**											**<0.0001**
Never used	931	22.4	240	26.9	279	24.4	217	19.5	195	19.5	
Current user	1,543	37.2	266	29.9	392	34.3	448	40.2	437	43.7	
Former user	1,324	31.9	319	35.8	376	32.9	352	31.6	277	27.7	
Unknown	353	8.5	66	7.4	97	8.5	98	8.8	92	9.2	
**BMI**											**0.001**
Underweight	167	4.0	26	2.9	47	4.1	40	3.6	54	5.4	
Normal weight	1,278	30.8	259	29.1	323	28.2	340	30.5	356	35.6	
Overweight	1,182	28.5	257	28.8	346	30.2	318	28.5	261	26.1	
Obese	1,031	24.8	243	27.3	303	26.5	271	24.3	214	21.4	
Unknown	493	11.9	106	11.9	125	10.9	146	13.1	116	11.6	
**Stage Summary**											0.31
Localized	2,113	50.9	474	53.2	600	52.5	567	50.9	472	47.2	
Regional	1,231	29.7	251	28.2	325	28.4	327	29.3	328	32.8	
Distant	628	15.1	125	14.0	169	14.8	175	15.7	159	15.9	
NA/Unknown	179	4.3	41	4.6	50	4.4	46	4.1	42	4.2	
**Charlson Comorbidity Index**											**0.03**
0	1,494	36.0	301	33.8	408	35.7	387	34.7	398	39.8	
1	1,235	29.7	252	28.3	348	30.4	341	30.6	294	29.4	
≥2	1,422	34.2	338	37.9	388	33.9	387	34.7	309	30.9	
**Hepatitis B/C (diagnosis before or within 6 months of cancer dx)**											**<0.0001**
No	1,980	47.7	482	54.1	572	50.0	512	45.9	414	41.4	
Yes	2,171	52.3	409	45.9	572	50.0	603	54.1	587	58.6	
**Surgery**											**<0.0001**
No	3,301	79.5	663	74.4	882	77.1	907	81.4	849	84.8	
Yes	850	20.5	228	25.6	262	22.9	208	18.7	152	15.2	
**Chemotherapy**											0.15
No	2,390	57.6	490	55.0	650	56.8	667	59.8	583	58.2	
Yes	1,607	38.7	369	41.4	444	38.8	404	36.2	390	39.0	
Unknown	154	3.7	32	3.6	50	4.4	44	4.0	28	2.8	
**Radiation**											**0.0004**
No	3,368	81.1	691	77.6	912	79.7	920	82.5	845	84.4	
Yes	725	17.5	193	21.7	210	18.4	180	16.1	142	14.2	
Unknown	58	1.4	7	0.8	22	1.9	15	1.4	14	1.4	
**Neoadjuvant radiotherapy**											0.63
No	4,097	98.7	877	98.4	1,127	98.5	1,104	99.0	989	98.8	
Yes	54	1.3	14	1.6	17	1.5	11	1.0	12	1.2	
**Neoadjuvant systemic therapy**											**0.01**
No	3,930	94.7	826	92.7	1,075	94.0	1,066	95.6	963	96.2	
Yes	219	5.3	64	7.2	69	6.0	49	4.4	37	3.7	
Unknown	2	0.1	1	0.1	0	0.0	0	0.0	1	0.1	
**Delayed surgery (>90 days after dx)**											**<0.0001**
No	552	13.3	150	16.8	154	13.5	147	13.2	101	10.1	
Yes	291	7.0	75	8.4	99	8.7	63	5.7	54	5.4	
Unknown	3,308	79.7	666	74.8	891	77.9	905	81.2	846	84.5	

Bold text indicates a p-value <0.05.

### 3.1 Incidence

The average annual liver cancer incidence rate from 2013 to 2017 in Louisiana was 9 cases per 100,000 population. The average (SD) CT AAIR was 8.02 (7.05) per 100,000 population (**Table 2**). The mean (SD) AAIR increased from 5.80 (4.75) in the least deprived Q1 to 9.26 (7.88) in Q4. The percentage of patients diagnosed at localized stage decreased as disadvantage increased (Q1 53.2% vs. Q4 47.2%, *p* = 0.31). Moreover, a lower proportion of cases residing in high-deprivation areas received treatment, including surgery and radiation compared to lower-deprivation areas (*p* < 0.001).

**Table 2:** Mean (standard deviation) hepatocellular carcinoma age-adjusted incidence rate by census tract Area Deprivation Index quartile, Louisiana, 2011–2020.

DI Quartile	Incidence Rate*		
	*N*	Mean	SD
Overall	1,128	8.02	7.05
Q1 (the least deprived)	271	5.80	4.75
Q2	271	7.61	6.05
Q3	271	9.02	7.19
Q4 (the most deprived)	271	9.26	7.88
Missing/Unknown	44	10.52	12.74

*Rates are per 100,000 and age-adjusted to the 2000 US standard population.

### 3.2 Receipt of treatment

**Figure 1:**
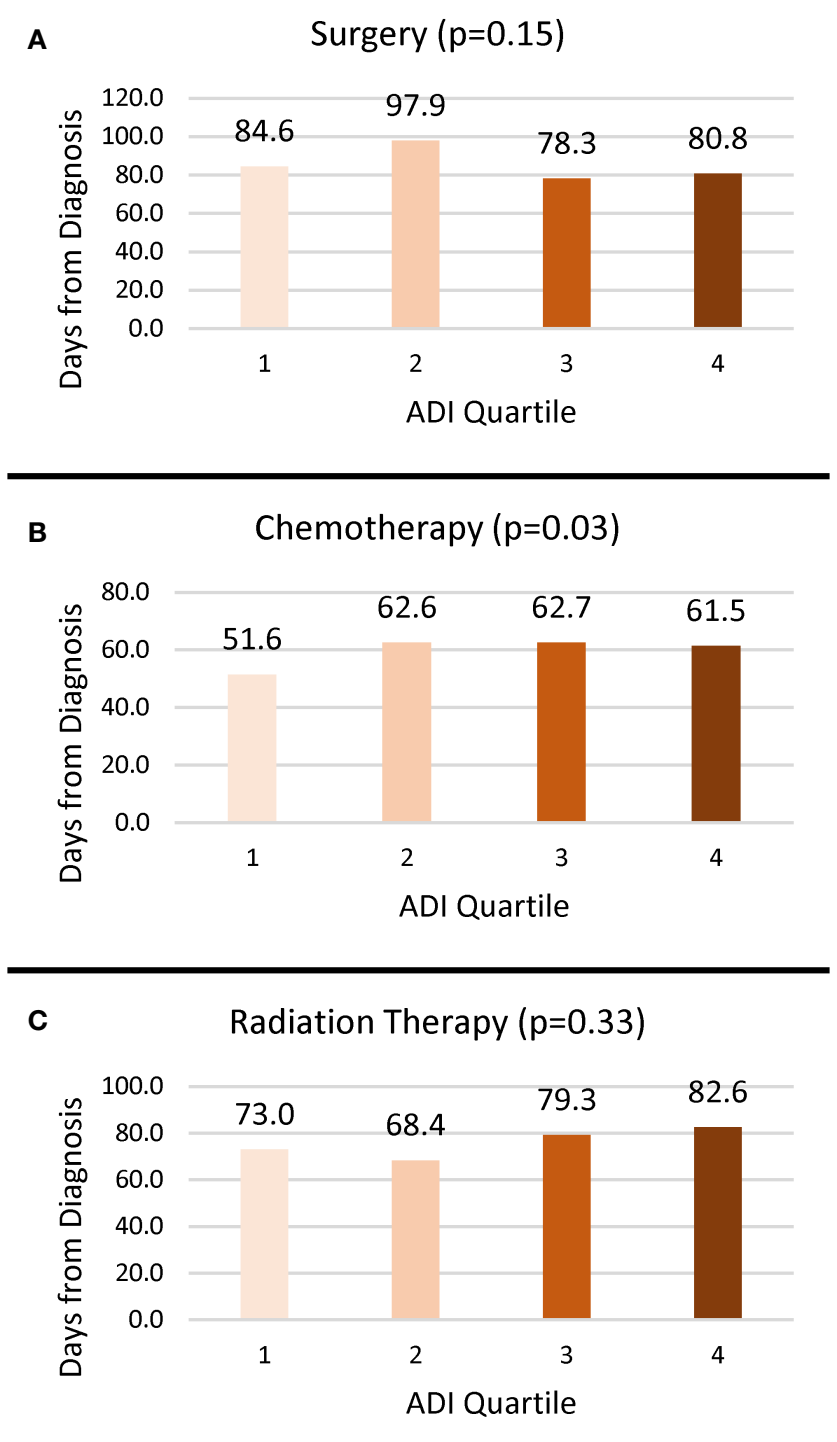
Mean number of days from diagnosis to treatment by ADI quartile. **(A)** Surgery. **(B)** Chemotherapy. **(C)** Radiation.

Overall, 20.5% of cases received surgery, 38.7% received chemotherapy, and 17.5% received radiation treatment (**Table 1**). The proportion of cases who received surgery (*p* < 0.0001) and radiation (*p* = 0.0004) decreased as deprivation increased, but ADI quartile was not associated with receipt of chemotherapy (*p* = 0.15). The time from diagnosis to initiation of treatment varied widely as evidenced by the large standard deviations. Delayed surgery, defined as more than 90 days after diagnosis, was associated with ADI quartile such that a greater proportion of those in the less deprived Q1 (8.4%) and Q2 (8.7%) experienced delay compared to those in the more deprived Q3 (5.7%) and Q4 (5.4%). Among those who received treatment, there was no significant difference in the average time from diagnosis to treatment for surgery (*p* = 0.15) and radiation (*p* = 0.33) based on ADI quartile (**Figure 1**). However, for those receiving chemotherapy, people living in Q1 CTs began treatment an average of 51.6 (SD 58.6) days from diagnosis, which was approximately 10 days earlier than those living in Q2 (62.6, SD 63.9), Q3 (62.7, SD 62.0), and Q4 (61.5, SD 55.6).

### 3.3 Survival

**Figure 2:**
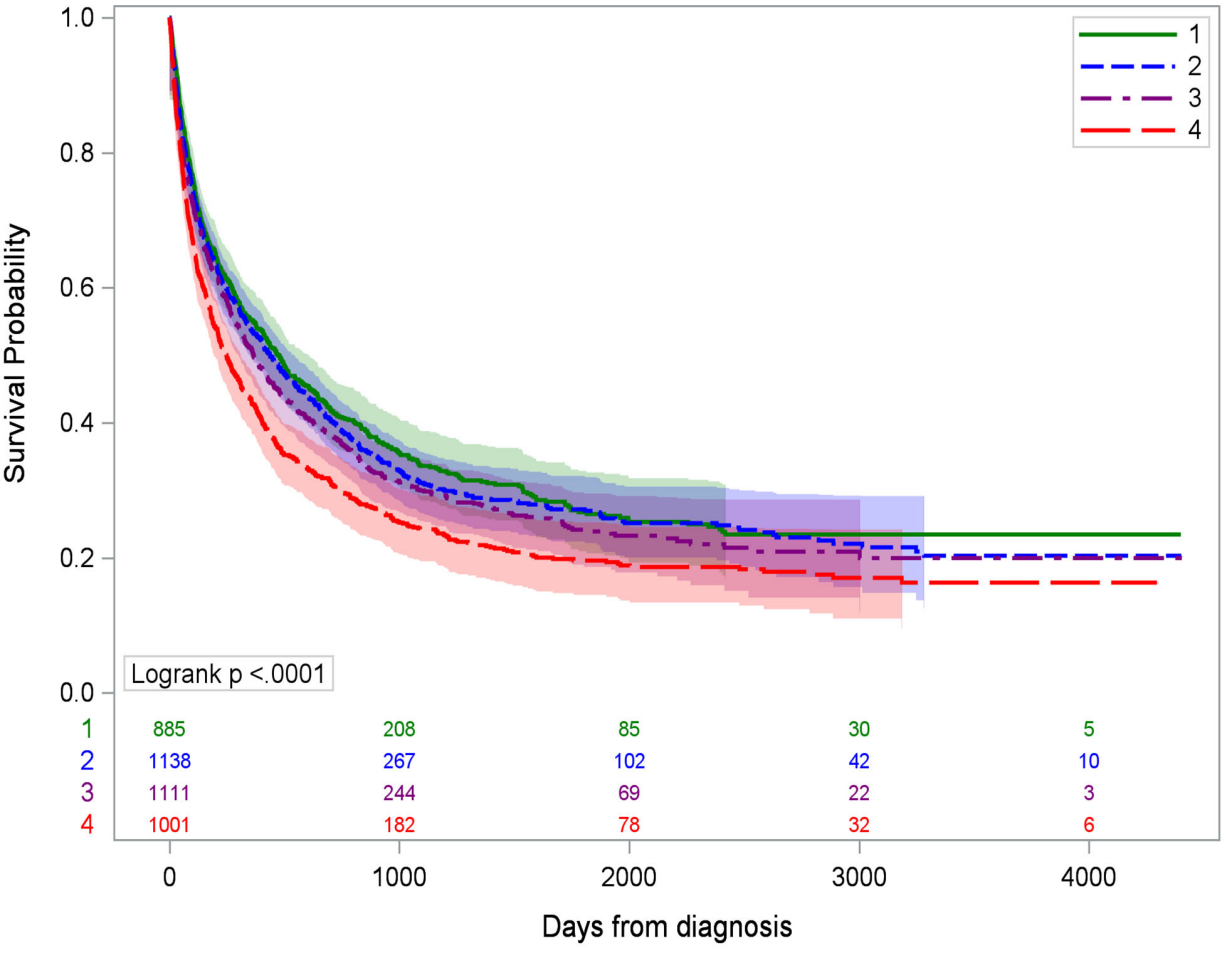
Kaplan–Meier survival curves for hepatocellular cancer-specific mortality by ADI quartile. Q1 is the least deprived and Q4 is the most deprived quartile of census tracts.

HCC patients residing in the most deprived CTs experienced the worst survival outcomes, with a median survival of 247 days (95% CI 211–290) compared to Q1 at 474 days (95% CI 407–547, *p* < 0.0001) (**Figure 2**). People with HCC living in the most deprived CTs had poorer survival (HR 1.20, 95% CI 1.05–1.37, *p* = 0.009) than Q1, but the association became attenuated (HR 1.12, 95% CI 0.96–1.30, *p* = 0.14) when adjusted for rurality, liquor store density, sex, race/ethnicity, age, insurance, BMI, stage, comorbidities, and history of hepatitis B/C infection (**Table 3**). In the adjusted model, hepatitis diagnosis increased the risk of death by 20% (HR 1.20, 95% CI 1.09–1.33) for Q4 vs. Q1 ADI. Living in a rural community, being male, being aged 60 or older, and using Medicaid or being uninsured were also associated with higher risk of HCC death. Diagnostic stage had an impact on survival, with regional stage HR 2.47 (95% CI 2.22–2.75), distant stage HR 4.47 (95% CI 3.92–5.09), and unknown stage HR 3.15 (95% CI 1.96–5.07) compared to localized stage. Compared to people with no comorbidities, those with one comorbidity were at decreased risk (HR 0.87, 95% CI 0.78–0.98) but the association for ≥2 comorbidities was not significant (HR 0.92, 95% CI 0.82–1.03). BMI, smoking status, race/ethnicity, and liquor store density were not statistically significant in the full model.

**Table 3:** Unadjusted and adjusted models for HCC-specific survival.

Variable	Unadjusted Models*				Adjusted Model			
	HR	95% CI		*p*-value	HR	95% CI		*p*-value
ADI quartile								
Q1 (least deprived)	Reference				Reference			
Q2	1.00	0.87	1.14	0.95	1.03	0.90	1.17	0.72
Q3	1.11	0.98	1.27	0.11	1.08	0.94	1.24	0.31
Q4 (most deprived)	1.20	1.05	1.37	**0.009**	1.12	0.96	1.30	0.14
Rural/urban status								
Urban commuting area	Reference				Reference			
Not an urban commuting area	1.13	0.98	1.30	0.09	1.19	1.03	1.39	**0.02**
**Liquor stores (per 1000 people)**	1.15	0.95	1.38	0.16	1.10	0.90	1.33	0.36
Gender								
Male	Reference				Reference			
Female	0.77	0.68	0.87	**<0.0001**	0.78	0.69	0.89	**0.0002**
Race/Ethnicity								
Non-Hispanic White	Reference				Reference			
Non-Hispanic Black	1.12	1.02	1.23	**0.02**	0.98	0.88	1.09	0.68
Hispanic	0.87	0.62	1.22	0.43	0.86	0.61	1.21	0.39
Age								
<50	Reference				Reference			
50–59	1.19	0.91	1.56	0.21	1.22	0.93	1.61	0.15
60–69	1.29	0.99	1.68	0.06	1.32	1.01	1.74	**0.05**
70–79	1.40	1.06	1.85	**0.02**	1.65	1.23	2.22	**0.001**
≥80	1.68	1.23	2.30	**0.001**	2.20	1.58	3.06	**<0.0001**
Primary Payer								
Private Insurance	Reference				Reference			
Medicaid	1.42	1.22	1.65	**<0.0001**	1.32	1.12	1.54	**0.0007**
Insured other	1.29	1.13	1.47	**0.0001**	1.13	0.98	1.29	0.10
Insurance Status Unknown	1.15	0.92	1.44	0.21	1.09	0.87	1.37	0.44
Not Insured	2.00	1.61	2.48	**<0.0001**	1.69	1.35	2.11	**<0.0001**
Smoking status								
Never used	Reference				Reference			
Current user	1.31	1.16	1.48	<0.0001	1.14	1.00	1.31	0.05
Former user	1.07	0.94	1.22	0.30	1.01	0.88	1.15	0.89
Unknown/not stated/no smoking specifics provided	1.24	1.02	1.50	**0.03**	1.06	0.86	1.31	0.57
BMI								
Underweight	Reference				Reference			
Normal weight	0.85	0.66	1.10	0.22	0.90	0.70	1.16	0.41
Overweight	0.63	0.49	0.82	**0.0004**	0.78	0.60	1.02	0.06
Obese	0.66	0.51	0.85	**0.001**	0.83	0.64	1.08	0.16
Unknown	0.90	0.69	1.18	0.44	1.00	0.75	1.32	0.98
Stage								
Localized	Reference				Reference			
Regional	2.53	2.27	2.81	0.06	2.47	2.22	2.75	**<0.0001**
Distant	4.83	4.25	5.48	**<0.0001**	4.47	3.92	5.09	**<0.0001**
NA/Unknown	3.33	2.08	5.31	**<0.0001**	3.15	1.96	5.07	**<0.0001**
Charlson Comorbidity Index								
0	Reference				Reference			
1	0.80	0.72	0.90	**0.0001**	0.87	0.78	0.98	**0.02**
≥2	0.82	0.74	0.92	**0.001**	0.92	0.82	1.03	0.14
Hepatitis B or C diagnosis (prior to or within 6 months of cancer diagnosis)								
No	Reference				Reference			
Yes	1.18	1.08	1.29	**0.001**	1.20	1.09	1.33	**0.0002**

*Each unadjusted model was run separately with only the variable in column 1 predicting survival time.

Bold text indicates p<0.05.

## 4 Discussion

This study adds to the literature by assessing the impact of area-level deprivation while controlling for known sociodemographic and clinical factors, as well as controlling for potential confounding by alcohol retail density. In this population-based study of adults diagnosed with HCC in Louisiana, CT-level deprivation was associated with age-adjusted incidence of HCC and median survival time. However, the effect of ADI on HCC survival was no longer significant when adjusted for individual and clinical variables. This finding suggests that ADI is an upstream risk factor influencing HCC risk and may indirectly impact HCC survival. One possible mechanistic explanation is racial inequity in the “accessibility, acceptability, and/or utilization” of medications approved to treat HCV, which were introduced in 1997 (17). HCV treatments are expensive and require compliance with a treatment regimen that can last weeks to months. Only approximately 1 in 3 individuals with health insurance get treatment within a year of diagnosis of HCV, with disparities by race and insurance status such that Black individuals and those using Medicaid are less likely to receive treatment (18). Thus, SDOH are an important consideration in one of the leading risk factors for HCC.

At-risk populations, including racial/ethnic minorities, have increased rates of HCC and poorer outcomes due to individual and area-level disparities in risk factors, screening, and treatment (13, 19–23). Multilevel interventions are likely needed to address the racial and economic disparities that contribute to liver cancer incidence and survival, such as differences in access to general healthcare that may impact screening and treatment for upstream risk factors such as alcohol use, obesity, and injection drug use.

Our examination of the association between area deprivation and treatment receipt and timing had mixed results. Previous studies from diverse geographies and racial/ethnic compositions have examined nSES with incident HCC, using various indices to measure the construct (11, 12, 24, 25). Taken in combination, these studies and ours support the importance of neighborhood context on disparities in HCC incidence, but additional research is needed to clarify potential associations with receipt of treatment. One suggested explanation for disparities in time to treatment initiation is access to patient navigation to help coordinate appointments (19, 26, 27).

Study strengths include the use of high-quality SEER registry data linked to HIDD data to enrich the hepatitis B/C status, a common risk factor for liver cancer. Although we controlled for a variety of individual sociodemographic and clinical characteristics in our adjusted survival model, some of these measures were imperfect predictors of liver damage risk. For example, BMI does not necessarily predict fatty deposits on the liver, leading to metabolic dysfunction-associated fatty liver disease (MAFLD) or the more advanced metabolic dysfunction-associated steatohepatitis (MASH). We also did not have direct measures of alcohol use history. Previous studies suggested that concentration of alcohol retailers is associated with heavy alcohol consumption (28), that neighborhoods with a mostly African American population and low nSES tend to have greater concentration of alcohol retailers (6), and that concentration of alcohol retailers is associated with negative health consequences including liver problems (29). Although we looked at area-level alcohol retail outlet density, many of the census tracts did not have any alcohol retailers, which limited its utility as an area-level predictor. Another limitation was using the Charlson Comorbidity Index as a global indicator of comorbidities rather than looking at specific comorbidities such as type 2 diabetes that may have a more direct impact on liver disease. Although we reported treatment-related delay as part of our descriptive statistics, we did not include this in the final adjusted Cox proportional hazards model because it was not significant in bivariate analysis. Additionally, treatment modality is impacted by diagnostic stage, which was already included for adjustment and could have led to potential overadjustment if both had been included in the same model. Another limitation of this study is generalizability. While the results accurately reflect the socioeconomic distribution of Louisiana, the census tract ADI quartile distribution likely varies from state to state. However, we would expect similar patterns of disadvantage to impact other populations in the US, although the impacts of controlling for race/ethnicity would also likely vary based on local distributions.

## 5 Conclusion

People living in census tracts with higher deprivation (as measured by ADI quartile) had increased risk of incident HCC. Although those living in the most deprived quartile had the shortest average survival time, the hazard ratio was no longer significant when the model adjusted for individual-level sociodemographic and clinical risk factors.

## Data availability statement

The data analyzed in this study is subject to the following licenses/restrictions: The data that support the findings of this study are available on request from the Louisiana Tumor Registry. The data are not publicly available due to privacy or ethical restrictions. Requests to access these datasets should be directed to https://sph.lsuhsc.edu/louisiana-tumor-registry/data-usestatistics/data-request/.

## Ethics statement

The studies involving humans were approved by Louisiana State University Health Sciences Center - New Orleans. The studies were conducted in accordance with the local legislation and institutional requirements. The ethics committee/institutional review board waived the requirement of written informed consent for participation from the participants or the participants’ legal guardians/next of kin because the study used public health surveillance data.

## Author contributions

KR: Conceptualization, Methodology, Project administration, Writing – original draft, Writing – review & editing. TL: Data curation, Formal analysis, Methodology, Visualization, Writing – original draft, Writing – review & editing. M-CH: Conceptualization, Data curation, Methodology, Supervision, Writing – original draft, Writing – review & editing. LT: Writing – review & editing. EP: Conceptualization, Funding acquisition, Methodology, Writing – review & editing.
